# Reduced Versus Oxidized NAD ^+^ Precursors Drive Distinct Transcriptomic, Proteomic, and Metabolic Profiles in Hepatocytes

**DOI:** 10.1096/fj.202501925R

**Published:** 2026-02-17

**Authors:** Kasper T. Vinten, Bauke V. Schomakers, Simone Denis, Michel van Weeghel, Aldo Jongejan, Rob Ofman, Sander R. Piersma, Connie R. Jimenez, Georges E. Janssens, Rubén Zapata‐Pérez, Riekelt H. Houtkooper

**Affiliations:** ^1^ Laboratory Genetic Metabolic Diseases, Amsterdam UMC University of Amsterdam Amsterdam the Netherlands; ^2^ Amsterdam Gastroenterology, Endocrinology, and Metabolism Institute Amsterdam UMC Amsterdam the Netherlands; ^3^ Core Facility Metabolomics Amsterdam UMC Amsterdam the Netherlands; ^4^ Amsterdam Cardiovascular Sciences Institute Amsterdam UMC Amsterdam the Netherlands; ^5^ Epidemiology and Data Science, Amsterdam UMC University of Amsterdam Amsterdam the Netherlands; ^6^ Amsterdam Public Health Methodology Amsterdam UMC Amsterdam the Netherlands; ^7^ OncoProteomics Laboratory Amsterdam UMC, Location VUmc, Medical Oncology Amsterdam the Netherlands; ^8^ Group of Metabolism and Gene Regulation, UCAM HiTech Sport & Health Innovation Hub Universidad Católica de Murcia Murcia Spain; ^9^ Emma Center for Personalized Medicine Amsterdam UMC Amsterdam the Netherlands

**Keywords:** comparative analysis, hepatocytes, NAD^+^, NAD^+^ precursors

## Abstract

Nicotinamide adenine dinucleotide (NAD^+^) is a vital molecule, serving as a redox cofactor and the limiting substrate for numerous enzymes. NAD^+^ decline is a key feature of aging, while supplementation with NAD^+^ precursors can efficiently counteract aging traits and prevent age‐associated conditions in preclinical models. However, clinical translation remains challenging, likely due to the limited NAD^+^ boosting capacity of classical precursors, such as nicotinamide mononucleotide (NMN) and nicotinamide riboside (NR). This has brought attention to their reduced forms, reduced NMN (NMNH) and reduced NR (NRH), which are more potent NAD^+^ boosters but remain poorly characterized. Here, we performed a comprehensive comparative analysis using RNA sequencing, proteomics, and metabolomics on cultured murine hepatocytes treated with NMN, NMNH, NR, or NRH. Global metabolic profiling revealed that NRH and NMNH induced substantially broader metabolic alterations than NR and NMN, with NRH uniquely suppressing metabolites involved in energy metabolism. The pronounced metabolic effects were reflected at a transcriptional level, with reduced precursors triggering a significantly higher number of differentially expressed genes than oxidized ones. Shared differentially expressed genes between NMNH and NRH revealed upregulation of stress‐related glutathione‐*S*‐transferases (*Gsts*) which furthermore were reflected in our proteomic profiling. However, the upregulation of *Gsts* did not cause a depletion of glutathione or oxiglutathione, suggesting a pseudo‐stress response to reduced NAD^+^ precursors. Together, our data demonstrate that reduced NAD^+^ precursors are unique and distinct from the market‐available NAD^+^ precursors NR and NMN, not only as more potent NAD^+^ boosters, but also as compounds influencing a broader range of cellular processes.

## Graphical abstract

Comparative transcriptomic, proteomic, and metabolomic analyses of reduced (NRH, NMNH) versus oxidized (NR, NMN) NAD^+^ precursors reveal that NRH and NMNH induce broader and more potent transcriptomic, proteomic, and metabolomic changes. Although all precursors increase NAD^+^ pathway metabolites, reduced precursors uniquely elicit a pseudo‐stress response, characterized by strong upregulation of glutathione‐*S*‐transferases both on a transcript as well as on a protein level without corresponding depletion of glutathione, underscoring different cellular impact between oxidized and reduced NAD^+^ precursors.

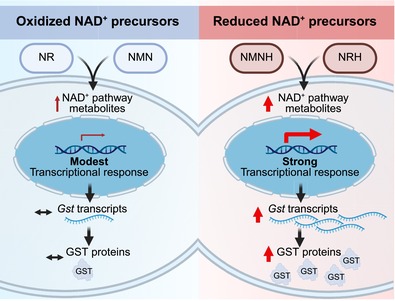

## 1 Introduction

Nicotinamide adenine dinucleotide (NAD^+^) serves as a metabolic hub connecting major cellular processes, including energy metabolism, where it serves as a redox cofactor, and as the limiting substrate for a plethora of regulatory enzymes [1]. Such enzymes include sirtuins (SIRTs), poly (ADP‐ribose) polymerases (PARPs), sterile alpha and Toll/interleukin‐1 receptor motif‐containing 1 (SARM1), and cyclic ADP‐ribose synthases, collectively known as NAD^+^ consumers [2]. As such, NAD^+^ can directly and indirectly influence cellular bioenergetics, DNA integrity, epigenetics, and inflammatory responses [3].

NAD^+^ levels gradually decline with age, and this has been linked to numerous inherited and age‐related conditions, such as neurodegenerative diseases [4, 5], metabolic diseases [6, 7], and age‐related physiological decline [8, 9, 10]. For this reason, strategies to replenish NAD^+^, like supplementation with NAD^+^ precursors, have attracted considerable attention, and have indeed proven effective in preventing or treating such diseases, at least in animal models [5, 6, 7, 9, 10, 11, 12, 13].

To date, a continuously growing panel of NAD^+^ boosters has been identified and characterized [14, 15]. Most notable are nicotinamide mononucleotide (NMN) and nicotinamide riboside (NR), which have been extensively studied in animal models and, to a lesser extent, in humans [16, 17, 18]. Both compounds have comparable NAD^+^ boosting capabilities, likely because they share a common pathway to NAD^+^. In fact, NMN requires extracellular conversion to NR prior to cellular uptake [19]. Once inside the cell, NR is phosphorylated back to NMN by NR kinases (NRKs) and finally converted to NAD^+^ via NMN adenylyltransferases (NMNATs) [20].

Despite the promising results obtained in preclinical models, NR and NMN have shown modest clinical efficacy [21, 22]. The underlying cause of such limited translatability remains to be fully elucidated but may, in part, be explained by considerable degradation of NR and NMN to nicotinamide (NAM) and nicotinic acid in the gastrointestinal tract and circulatory system [19, 23, 24, 25, 26, 27]. Furthermore, the NAD^+^ boosting potential of NMN and NR is limited to around two‐fold [6, 7].

Recently, a new class of NAD^+^ precursors has been identified: the reduced counterparts of NR and NMN, known as NRH and NMNH, respectively [28, 29, 30]. As with NR, only NRH can be transported across the cell membrane via specific nucleoside transporters, whereas NMNH requires dephosphorylation to NRH beforehand [28, 30]. Intracellularly, both NRH and NMNH are independent of NRKs, but rely on the phosphorylating activity of adenosine kinase for their conversion to NAD^+^ [28, 30, 31]. Both compounds are more potent NAD^+^ boosters compared to NR and NMN [28, 30, 31]. The potent effect of reduced precursors on NAD^+^ levels could potentially overcome some of the limitations associated with NR and NMN supplementation. However, as NRH and NMNH remain untested in humans, it is essential first to understand their biological effects to evaluate their translational potential and determine whether they can be used as an alternative strategy to raise NAD^+^ levels. To date, side‐by‐side comparative analyses of NAD^+^ precursors are restricted to the impact on the NAD^+^ metabolome, or the effects on isolated phenotypes.

In this study, we systematically evaluate and compare the downstream transcriptomic, proteomic, and metabolic profiles following supplementation with the NAD^+^ precursors NR, NMN, NRH, and NMNH in mouse hepatocytes. This work provides the first comprehensive, side‐by‐side comparison of the effects of the classical NAD^+^ precursors NR and NMN alongside the more recently identified and potent precursors NRH and NMNH under conditions of acute cellular exposure.

## 2 Materials and Methods

### 2.1 Cell Culture

AML12 murine hepatocytes were cultured in 6‐well plates for transcriptomics, metabolomics, and proteomics, or in T75 flasks for cytosolic mitochondrial DNA (mtDNA) determination. Cells were grown in Dulbecco's modified Eagle's medium (DMEM, Life Technologies) supplemented with 10% fetal bovine serum (FBS, BioWhittaker), 100 U/mL penicillin, and 10 μg/mL streptomycin. Cells were treated with PBS (vehicle) or 500 μM NMN, NR, NMNH, or NRH for 24 h prior to DNA, RNA, metabolite, or protein extraction. For cytosolic mtDNA measurements, cells treated with 1 μM doxorubicin for 24 h were included as an additional condition.

### 2.2 mRNA Isolation

RNA was isolated using TRIzol reagent according to standard protocols, and extracted according to the manufacturer's instructions for the RNeasy Mini Kit (QIAGEN). Contaminating genomic DNA was removed using RNase‐free DNase (QIAGEN). RNA was quantified with a NanoDrop 2000 spectrophotometer (Thermo Scientific; Breda, The Netherlands) and stored at −80°C until use.

### 2.3 RNA Sequencing

RNA libraries were prepared and sequenced with the Illumina platform by Genome Scan (Leiden, The Netherlands). The NEBNext Ultra II Directional RNA Library Prep Kit for Illumina (#E7760S/L) was used to process the samples according to the manufacturer's instructions. Briefly, mRNA was isolated from total RNA using the oligo‐dT magnetic beads. After mRNA fragmentation, cDNA synthesis was performed. This was used for ligation with the sequencing adapters and PCR amplification of the resulting product. The quality and yield after sample preparation were measured using a Fragment Analyzer. The size of the resulting products was consistent with the expected size distribution (a broad peak between 300 and 500 bp). Clustering and DNA sequencing using the NovaSeq6000 were performed according to the manufacturer's protocols. A concentration of 1.1 nM of DNA was used. NovaSeq control software (NCS) v1.6 was used.

### 2.4 Metabolomics

Metabolomics was performed as previously described [32]. Briefly, PBS‐washed cells were metabolically quenched by adding 500 μL ice‐cold methanol. The following internal standards dissolved in 500 μL Milli‐Q water were added: D_5_‐glutamine, D_5_‐monophosphate, adenosine‐^15^N_5_‐triphosphate, and guanine‐^15^N_5_‐triphosphate (5 μM each). 1 mL chloroform was added to all samples followed by thorough mixing. Samples were centrifuged at 16000 rpm for 5 min at 4°C and the upper aqueous phase containing polar metabolites transferred to new Eppendorf tubes. Samples were concentrated to dryness in a vacuum concentrator (90°C) and pellets reconstituted in 100 μL of 3:2 methanol:Milli‐Q water (v/v) prior to mass spectrometry analysis. Metabolite analysis was carried out on an Acquity UPLC system (Waters, Milford, MA, USA) coupled to an Impact II Ultra‐High Resolution Qq‐Time‐Of‐Flight mass spectrometer (Bruker). Chromatographic separation of the compounds was achieved using a SeQuant ZIC‐cHILIC column (PEEK 100 × 2.1 mm, 3 μm particle size; Merck, Kenilworth, NJ, USA) at 30°C. The LC method consisted of a gradient running at 0.25 mL/min from 100% mobile phase B (9:1 acetonitrile: water with 5 mM ammonium acetate, pH 8.2) to 100% mobile phase A (1:9 acetonitrile: water with 5 mM ammonium acetate, pH 6.8) over 28 min, followed by a re‐equilibration step at 100% B for 5 min. MS data were acquired in both negative and positive ionization modes over the *m*/*z* range of 50–1200. Data from full‐scan MS mode were analyzed using Bruker TASQ software version 2021b (2021.1.2.452). All reported metabolite intensities were normalized to total protein content in the samples, determined with a Pierce BCA Protein Assay Kit, as well as to internal standards with comparable retention times and MS response. Statistical analyses were performed using the edgeR version 4.2.2 [33], and limma/voom version 3.60.6 [34] R packages. Relative abundances were transformed to log_2_, normalized by applying the trimmed mean of *M*‐values method [33] and precision weighted using voom [35]. Statistical significance was assessed using an empirical Bayes moderated *t*‐test within limma's linear model framework including the precision weights estimated by voom [34, 35].

### 2.5 RNA Sequencing, Read Mapping, and Statistical Analyses

Reads were subjected to quality control using FastQC [36] and dupRadar version 1.0.0 [37], trimmed using Trimmomatic version 0.32 [38] and aligned using HISAT2 version 2.1.0 [39] against the murine genome (GRCm38.v93). Counts were obtained using HTSeq (version 0.11.0, default parameters) [40] using the corresponding GTF (v93) taking into account the directions of the reads. Statistical analyses were performed using the edgeR version 3.26.8 [33] and limma/voom version 3.40.6 [34] R packages. All genes with more than 2 counts in at least 2 of the samples were kept. Count data were transformed to log_10_‐counts per million (logCPM), normalized by applying the trimmed mean of M‐values method [33], and precision weighted using voom [35]. Differential expression was assessed using an empirical Bayes moderated *t*‐test within limma's linear model framework including the precision weights estimated by voom [34, 35]. Data processing was performed using R v3.6.1 and Bioconductor version 3.9. Genes were re‐annotated using biomaRt using the Ensembl genome databases (version 99).

### 2.6 Proteomics

PBS‐washed cells were metabolically quenched by adding ice‐cold methanol. After thorough mixing, samples were centrifuged for 10 min at 14000 rpm and the resulting pellet was dried under a gentle stream of nitrogen. Proteomics sample preparation was performed using a Thermo Scientific EasyPep MS Sample Prep Kit (A40006), according to the manufacturer's instructions. Briefly, 200 μL lysis buffer was added to each sample and a Thermo Scientific Pierce BCA Protein Assay (23225) was performed according to manufacturer's instructions to determine protein content. For each sample, 100 μg of protein was transferred to a new 2 mL Eppendorf tube. Samples were reduced and alkylated at 95°C for 10 min, followed by a 2‐hour incubation at 37°C with a Trypsin/Lys‐C protease mixture. After sample clean‐up with the Peptide Clean‐up Plate, samples were dried under nitrogen at 40°C, before being resuspended in a 100 μL mixture of 97:3 (v/v) water: acetonitrile, containing 0.1% formic acid. Samples were kept at 12°C during analysis and 10 μL of each sample was injected. Injection order for samples was random, with injections of a pooled sample at the start and end, as well as at varying intervals throughout the series. Chromatographic separation was achieved on a Waters ACQUITY Premier UPLC (quaternary solvent manager, bioinert), using a Waters ACQUITY Premier UPLC BEH C18 Column (1.7 μm, 2.1 mm × 50 mm) (186009452). Column temperature was held at 60°C. Mobile phase consisted of (A) water and (B) acetonitrile, both containing 0.1% formic acid. Using a starting flow rate of 0.5 mL/min, the LC gradient consisted of: dwell at 3% B for 0–0.1 min; ramp to 40% B at 4.3 min; ramp to 80% B at 4.31 min with a flow rate of 0.85 mL/min; dwell at 80% B for 4.31–4.40 min with a flow rate of 0.85 mL/min; ramp to 3% B at 4.50 min with a flow rate of 0.6 mL/min; dwell at 3% B for 4.5–5 min with a flow rate of 0.5 mL/min. MS data were acquired with a Bruker timsTOF Pro 2 using positive ionization in DIA‐PASEF mode as previously reported [41]. DIA‐PASEF data files were processed using DIA‐NN version 1.8.1 [42], using an empirically generated spectral library for human proteins provided by Bruker Daltonics. The following DIA‐NN options were enabled: Reannotate Contaminants, N‐term M excision, C carbamidomethylation, MBR, No shared spectra. Other settings were as follows: Missed cleavages: 1; Peptide length range: 7–30; Precursor charge range: 1–4; Precursor *m*/*z* range: 300–1800; Fragment ion *m*/*z* range: 200–1800; Precursor FDR (%): 1; Mass accuracy: 0.0; MS1 accuracy: 0.0; Scan window: 0; Protein names (from FASTA); Double‐pass mode; Quant UMS (high precision); RT‐dependent; IDs, RT & IM profiling; Optimal results. Data were normalized in DIA‐NN using MaxLFQ [43]. Statistical analyses were performed using the edgeR version 4.2.2 [33] and limma/voom version 3.60.6 [34] R packages. Relative abundances were transformed to log_2_, normalized by applying the trimmed mean of *M*‐values method [33], and precision weighted using voom [35]. Statistical significance was assessed using an empirical Bayes moderated *t*‐test within limma's linear model framework including the precision weights estimated by voom [34, 35].

### 2.7 Cytosolic mtDNA Determination

Cellular fractionation for cytosolic mtDNA determination was performed according to a previously described protocol, with modifications [44]. Briefly, PBS‐washed cells were resuspended in digitonin lysis buffer (200 mM mannitol, 4 mM Tris, 0.4 mM EDTA, pH 7.4, and 20 μg/mL digitonin) and left on ice for 10 min to allow plasma membrane permeabilization followed by centrifugation at 3000 *g* for 3 min at 4°C. The supernatant was transferred to a new Eppendorf tube and centrifuged at 14000 *g* for 5 min at 4°C to isolate the cytosolic fraction, separating it from nuclei, mitochondria, and other organelles. The pellet was resuspended in a buffer containing 250 mM mannitol, 5 mM Tris, 0.5 mM EDTA, pH 7.4. DNA was then extracted from the cytosolic and the whole cell lysate fraction using the QIAamp DNA Mini Kit (QIAGEN) according to the manufacturer's instructions followed by quantification of DNA amount with a NanoDrop 2000 spectrophotometer (Thermo Scientific; Breda, The Netherlands). Quantitative PCR was performed on both the cytosolic and whole cell fractions using mtDNA primers: *Cox2* (fwd: GTTGATAACCGAGTCGTTCTGC, rev: CCTGGGATGGCATCAGTTTT) and *16 s* (fwd: CCGCAAGGGAAAGATGAAAGAC, rev: TCGTTTGGTTTCGGGGTTTC). The relative cytosolic mtDNA levels were normalized to whole cell mtDNA amounts.

### 2.8 Generative Artificial Intelligence

ChatGPT‐4 and ChatGPT‐5 were used during the preparation of this work to support text refinement and code development. All AI‐assisted content was subsequently reviewed, edited, and approved by the authors, who take full responsibility for the final publication.

### 2.9 Data Processing and Visualization

**FIGURE 1:**
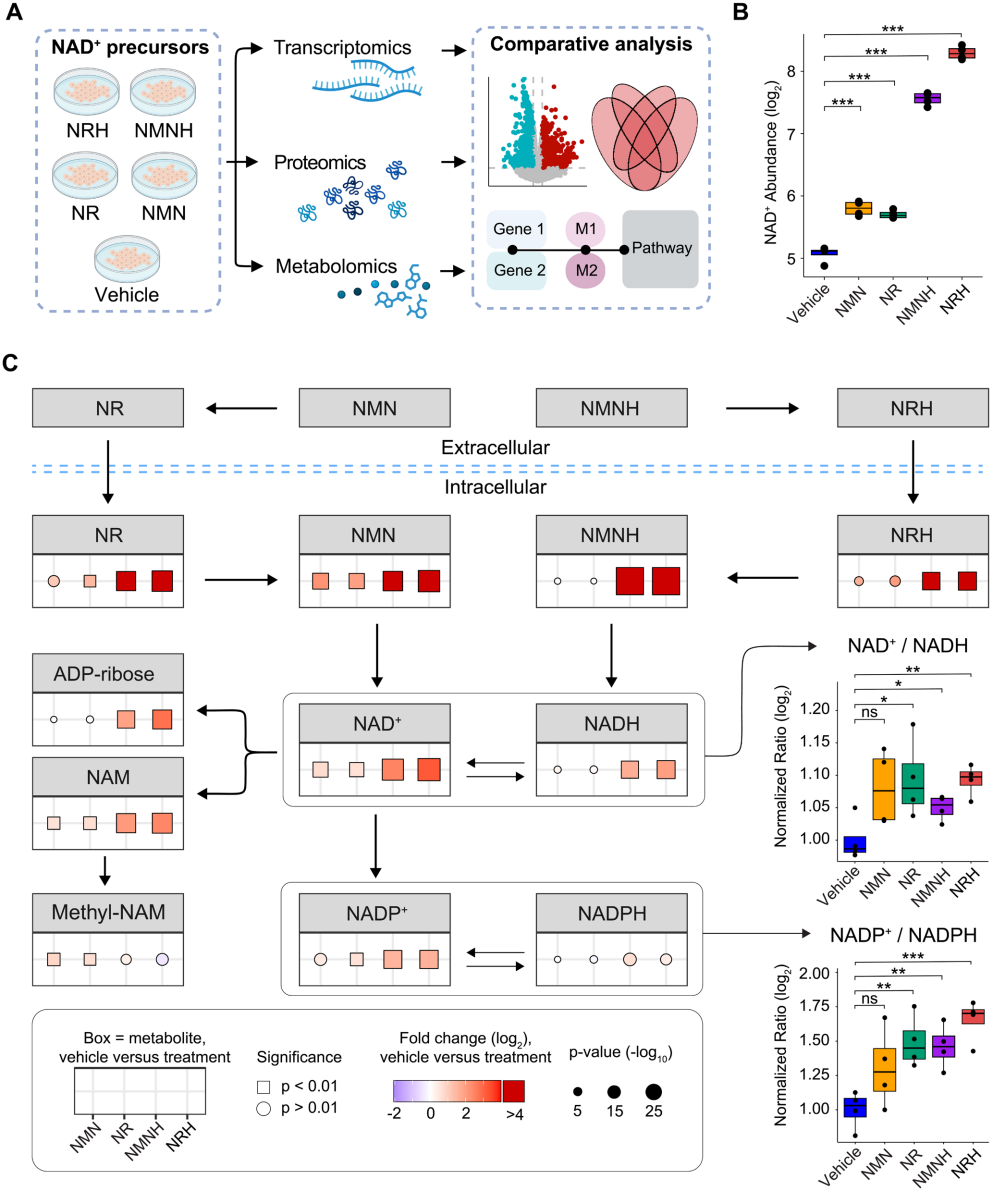
Effect of oxidized and reduced NAD^+^ precursors on the NAD^+^ metabolome. (A) Study flowchart. Murine AML12 cells were incubated for 24 h in the presence of NRH, NMNH, NR, NMN (500 μM), or vehicle (PBS) (*n* = 3–4 per group). Cell extracts were collected and analyzed using UHPLC–MS or RNAseq. (B) NAD^+^ levels following NAD^+^ precursor treatment. (C) NAD^+^ precursor effect on the NAD^+^ metabolome relative to the vehicle‐treated group. Shape, color, and size indicate significance threshold (square: *P* < 0.01; circle: *P* > 0.01), fold change relative to vehicle (log_2_, blue: Decreased; red: Increased) and *p*‐value (−log_10_), respectively. Empirical Bayes moderated *t*‐test (two‐sided, unadjusted *p*‐values) was used to determine statistical significance, *p < 0.05, **p < 0.01, ***p < 0.001, ns = not significant. Normalized redox ratios of log_2_ abundances were calculated relative to the vehicle group and analyzed using one‐way analysis of variance (ANOVA). Whisker plots display the median, interquartile range (box edges from 25th to 75th percentile), whiskers extending up to 1.5× interquartile range, and individual outliers beyond this range. Source data is available in Table S1.

Data processing was performed using R version 4.4.1 and Bioconductor version 3.19. Data were processed in part with the R package dplyr version 1.1.4 [45], tidyverse 2.0.0 [46], and PerformanceAnalytics version 2.0.8 [47]. Biological process overrepresentation analysis was performed using clusterProfiler version 4.12.6 [48] and org.Mm.eg.db version 3.19.1 [49]. Data was visualized using ggplot2 version 3.5.1 [50], ggrepel version 0.9.6 [51], VennDiagram version 1.7.3 [52], pheatmap version 1.0.12 [53], RColorBrewer version 1.1.3 [54], and viridis version 0.6.5 [55]. Illustrations in Figure 1A and the graphical abstract were generated using BioRender.com.

## 3 Results

### 3.1 Reduced NAD ^+^ Precursors Are Potent Enhancers of the NAD ^+^ Metabolome

Using transcriptomic, proteomic, and metabolomic profiling, we assessed the downstream effects of NAD^+^ precursors in AML12 murine hepatocytes supplemented with NR, NMN, NRH, or NMNH (Figure 1A). All precursors were supplemented at a dose of 500 μM, which is the lowest dose that gives the highest NAD^+^ boost in hepatocytes and has proven therapeutic benefits in in vitro models of kidney injury [28, 30].

Consistent with previous findings, supplementation with the reduced NAD^+^ precursors NRH and NMNH caused a potent increase in NAD^+^ levels, largely surpassing that of the oxidized precursors NR and NMN (Figure 1B). To elucidate the effects of the different NAD^+^ precursors on the NAD^+^ metabolome, we outlined the biosynthetic and degradation pathway‐related metabolites from each precursor (Figure S1A–L) and calculated their change relative to the vehicle group (Figure 1C). NRH and NMNH significantly increased NADH levels, while NR or NMN failed to do so. ADP‐ribose, a reaction product of NAD^+^ consuming enzymes, was significantly increased following NRH or NMNH treatment, while unaffected following NR or NMN treatment. In contrast, NAM, another reaction product of NAD^+^ consumers, increased in all groups. Methyl‐NAM, a direct degradation product of NAM, remained unaltered following NRH or NMNH treatment, but was significantly more abundant following treatment with NR or NMN (Figure 1C). Oxidized NAD^+^ precursors are converted to NAD^+^, whereas reduced precursors are presumed to initially generate NADH, which is subsequently oxidized to NAD^+^ by cellular dehydrogenases. Despite these different entry routes, all precursors led to a comparable increase in the cellular NAD^+^/NADH ratio (Figure 1C, Figure S2A). A similar pattern was observed in the ratio of their phosphorylated counterparts, NADP^+^/NADPH (Figure 1C, Figure S2B). Overall, these results confirm that NRH and NMNH supplementation has a more profound impact on the NAD^+^ metabolome than NR and NMN supplementation, though the influence on the NAD^+^/NADH and NADP^+^/NADPH ratios is less pronounced.

### 3.2 Reduced NAD ^+^ Precursors Elicit Strong but Divergent Metabolic Responses

**FIGURE 2:**
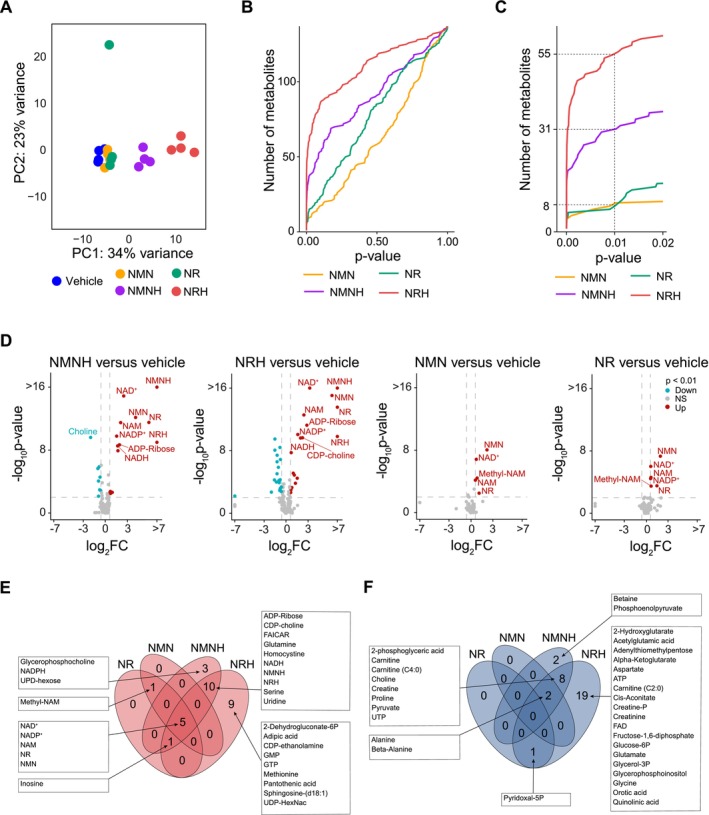
Metabolic profiling following NAD^+^ precursor treatment. (A) Principal component analysis (PCA) of AML12 cells treated with NRH, NMNH, NR, NMN (500 μM), or vehicle (PBS) (*n* = 4 per group). (B) Distribution of metabolites based on *p*‐values across all measured metabolites and (C) number of statistically significant metabolites at *p* = 0.01, indicated by intersections for each treatment. (D) Volcano plots representing metabolite changes following NAD^+^ precursor treatment. The *x* axis represents fold change (log_2_), and the *y* axis represents *p*‐value (−log_10_). Color indicates metabolites significantly more abundant (red), less abundant (blue), or not significant (NS) relative to the vehicle group. Dashed lines mark ±0.6 fold change (log_2_) and *p* = 0.01 on the *x* and *y* axes, respectively. The top 10 most significant metabolites are annotated. (E, F) Venn diagrams of (E) accumulated (red) and (F) depleted (blue) metabolites following NAD^+^ precursor treatment (*p* < 0.01). The metabolites in each intersection are shown in text boxes. Empirical Bayes moderated *t*‐test (two‐sided, unadjusted *p*‐values) was used to determine statistical significance. Source data is available in Table S1.

After confirming the effects of oxidized and reduced NAD^+^ precursors on the NAD^+^ metabolome, we next asked what broader metabolic changes were triggered by supplementation with each precursor (Figure 2). A total of 137 metabolites were detected using ultra‐high‐performance liquid chromatography (UHPLC) coupled to high‐resolution mass spectrometry (MS). To evaluate treatment‐specific alterations in the metabolome, we performed principal component analysis (PCA) for multidimensional reduction of the data. We found that NMNH and NRH clustered distantly from the vehicle group along principal component 1, whereas NMN and NR remained closer to the vehicle group (Figure 2A). Out of the 137 annotated metabolites, 8 were significantly changed after NMN and NR supplementation in comparison with vehicle‐treated cells, while NMNH and NRH triggered statistically significant changes in a total of 31 and 55 metabolites, respectively (Figure 2B,C). Volcano plots of the metabolic changes after administration with the different precursors showed that the reduced compounds exerted a stronger impact on the metabolome compared to their oxidized counterparts, with the most pronounced changes observed in key NAD^+^‐related metabolites, including NMN, Methyl‐NAM, NAM, NR, and NAD^+^ (Figure 2D).

Next, we determined which metabolites were commonly affected across treatments (Figure 2E,F). Only a limited subset of metabolites was shared between NR and NMN, as well as between oxidized and reduced NAD^+^ precursors. In fact, only NAD^+^‐related metabolites were significantly more abundant upon supplementation with any of the oxidized or reduced precursors. Elevated levels of metabolites in the intersection between NMNH and NRH included nucleotide‐related metabolites such as ADP‐ribose, FAICAR, uridine, and CDP‐choline. Additionally, other nucleotides, such as GMP, GTP, and UDP‐N‐acetylhexosamine (UDP‐HexNac), were exclusively elevated after NRH supplementation (Figure 2E). Moreover, several metabolites related to energy metabolism, such as the glycolytic intermediate, glucose‐6‐phosphate, the TCA cycle intermediates cis‐aconitate and alpha‐ketoglutarate, as well as ATP, were found less abundant solely after NRH supplementation, suggesting that NRH suppresses energy metabolism (Figure 2F). Of note, the glycolytic intermediate 2‐phosphoglyceric acid and the end product of glycolysis, pyruvate, were less abundant in the intersection between NRH and NMNH.

Collectively, these results show that, while all NAD^+^ precursors influence the NAD^+^ metabolome, reduced precursors, particularly NRH, trigger more profound metabolic changes, including a potential suppression of energy metabolism. This suggests distinct effects not only between oxidized and reduced precursors but also between NRH and NMNH.

### 3.3 Reduced NAD ^+^ Precursors Impart Transcriptional Changes That Reach Beyond NAD ^+^‐Related Genes

**FIGURE 3:**
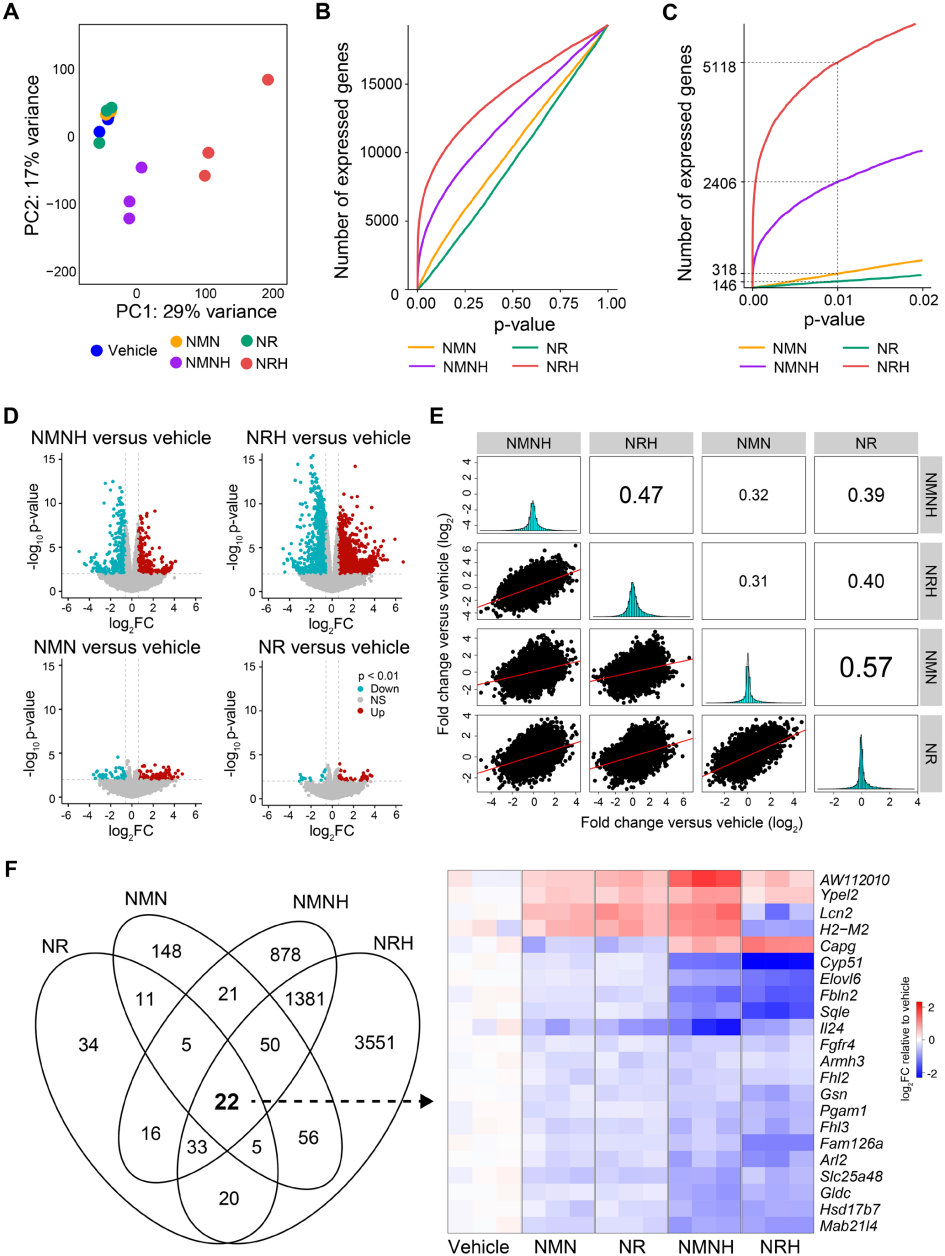
Transcriptomic profiling following NAD^+^ precursor treatment. (A) Principal component analysis (PCA) of AML12 cells treated with NRH, NMNH, NR, NMN (500 μM), or vehicle (*n* = 3 per group). (B) Distribution of transcripts based on *p*‐values across all measured transcripts and (C) number of statistically significant transcripts at *p* = 0.01 indicated by intersections for each treatment. (D) Volcano plots representing transcripts following NAD^+^ precursor treatment. The *x* axis represents the fold change (log_2_), and the *y* axis represents *p*‐value (−log_10_). Color indicates upregulated (red), downregulated (blue), or not significant (NS) genes relative to the vehicle group. Dashed lines mark ±0.6 fold change (log₂) and *p* = 0.01 on the *x* and *y* axes, respectively. (E) Pairwise correlations of transcriptomic responses between each NAD^+^ precursor based on their fold change relative to the vehicle group. The scatterplot matrix illustrates the correlation between each NAD^+^ precursor. The diagonal panels show the distribution of fold change values for each condition. Correlation coefficients (Pearson's *r*) are indicated for each pairwise comparison. (F) Venn diagram of overlapping significant transcripts (*p* < 0.01) between each treatment group. Uniquely shared transcripts between all NAD^+^ precursors are presented in a heatmap with the upregulated (red) and downregulated (blue) differentially expressed genes normalized to the vehicle group. Empirical Bayes moderated *t*‐test (two‐sided, unadjusted *p*‐values) was used to determine statistical significance. Source data is available in Table S2.

Having established that reduced NAD^+^ precursors induce distinct metabolic changes compared to their oxidized equivalents, we next asked whether the alterations observed in the metabolome would be reflected at a transcriptional level. To explore this, we performed RNA sequencing to compare gene expression changes across treatments. Similar to what we observed at a metabolic level, PCA showed a clear separation between oxidized and reduced NAD^+^ precursors, with oxidized precursors exhibiting a greater similarity to the vehicle group (Figure 3A). This separated clustering of reduced precursors was reflected with a substantially greater number of transcriptional changes (Figure 3B). Specifically, using a *p*‐value of 0.01 as cut‐off, 146 and 318 genes were differentially expressed following treatment with NR or NMN, respectively, while 2406 and 5118 genes were differentially expressed following treatment with NMNH or NRH, respectively (Figure 3C). The differentially expressed genes were approximately equally distributed between up‐ and downregulated genes for each NAD^+^ precursor compared to the vehicle‐treated group (Figure 3D).

Next, we investigated the transcriptional relationships between each NAD^+^ precursor. To do so, we made pairwise comparisons of the fold change of gene expression between the different NAD^+^ precursor treatments relative to the vehicle group (Figure 3E). Each scatter plot represents the correlation of fold changes across all expressed genes, with Pearson correlation coefficients shown in the upper right quadrants and histograms visualizing the distribution of fold change values for each treatment compared to the vehicle group (Figure 3E). We found Pearson correlations between oxidized (NMN and NR, *r* = 0.57) and reduced precursors (NMNH and NRH, *r* = 0.47) to be the highest among all correlations, suggesting that each of the precursor pairs induces related transcriptional changes.

To identify the unique transcripts across all treatments, we next performed Venn diagram analysis and isolated genes significantly regulated by all four compounds (NMN, NR, NMNH, and NRH) compared to the vehicle group (Figure 3F). In total, 22 genes were identified at the intersection between all treatments. Although all precursors effectively enhanced NAD^+^ levels, none of the commonly regulated genes were directly involved in NAD^+^ metabolism. Nonetheless, two metabolic‐ and mitochondria‐related genes, namely glycine decarboxylase (*Gldc*), involved in mitochondrial one‐carbon metabolism [56], and solute carrier family 25 member 48 (*Slc25a48*), a mitochondrial choline transporter [57], were downregulated across all treatments.

Given their broad and distinct transcriptional impact on genes beyond NAD^+^‐related genes, reduced NAD^+^ precursors may influence a wide range of cellular pathways. This provides opportunities for both therapeutic benefits as well as potentially detrimental effects associated with reduced NAD^+^ precursors.

### 3.4 Reduced NAD ^+^ Precursors Upregulate Glutathione‐*S*‐Transferases Without Impacting Glutathione Levels

**FIGURE 4:**
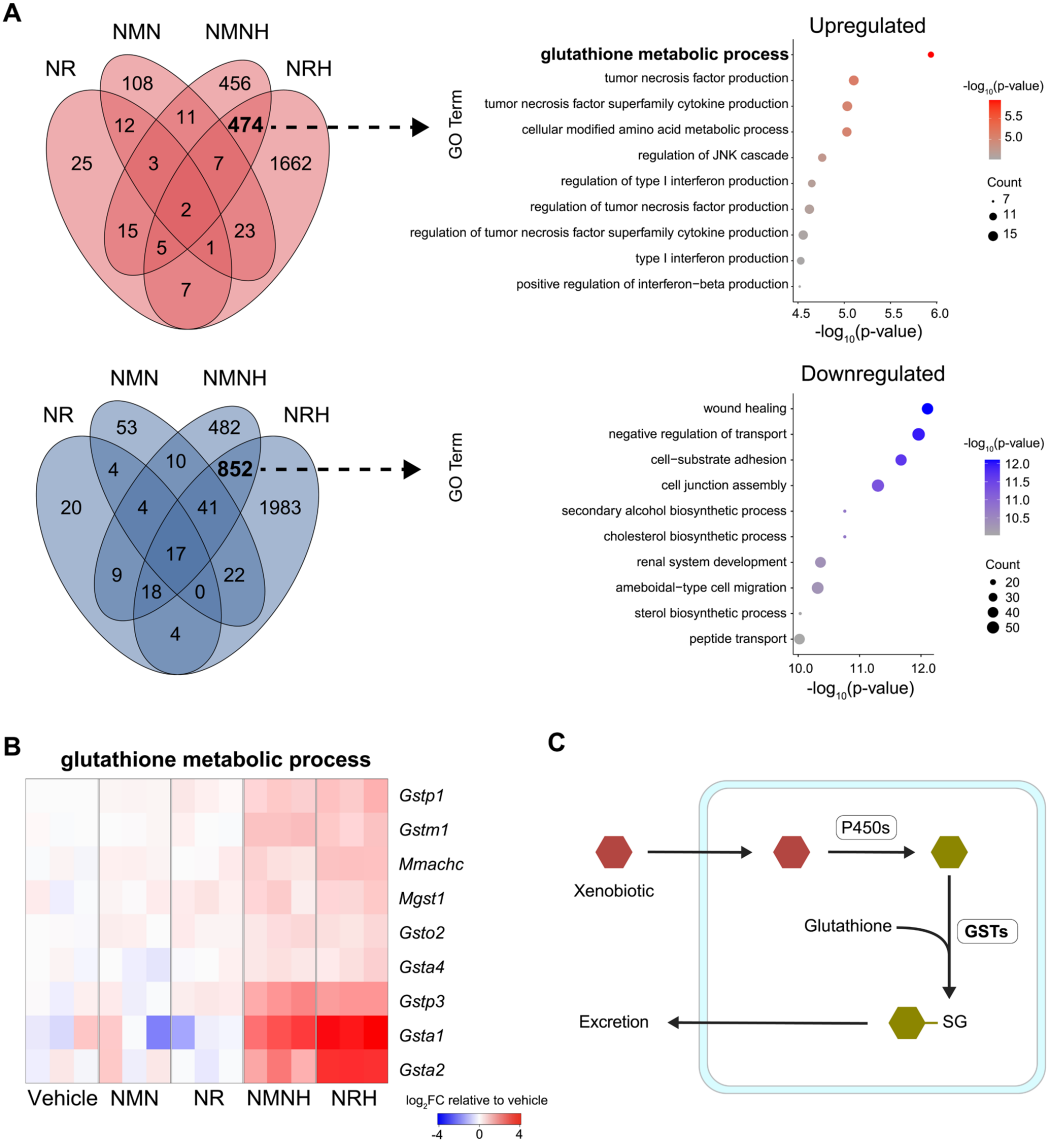
Glutathione‐related transcripts following NRH and NMNH treatment. (A) Venn diagrams of upregulated (red) and downregulated (blue) genes following NAD^+^ precursor treatment. Gene ontology (GO) term analysis has been applied to the intersections between the reduced NAD^+^ precursors NRH and NMNH. (B) Heatmap displaying genes extracted from the GO term, *glutathione metabolic process*. Color represents the magnitude of upregulation (red) or downregulation (blue), normalized to the vehicle group. (C) Schematic illustrating detoxification of an arbitrary xenobiotic, initiated by cytochrome P450 enzymes (P450), followed by glutathione conjugation by a glutathione‐*S*‐transferase (GST), leading to the formation of a glutathione conjugate designated for excretion.

Given the strong transcriptional response elicited by reduced NAD^+^ precursors, we next aimed to study the differentially expressed genes shared by NRH and NMNH. We divided the transcriptional‐based Venn diagram into up‐ and downregulated genes and found that 474 upregulated and 852 downregulated genes were shared between NMNH and NRH. Additionally, NMNH and NRH individually upregulated the expression of 456 and 1662 genes and downregulated the expression of 482 and 1983 genes, respectively (Figure 4A). Gene ontology (GO) enrichment analysis of upregulated genes in the intersection between NRH and NMNH revealed a significant overrepresentation of the GO term, *glutathione metabolic process*, driven primarily by genes encoding glutathione‐*S*‐transferases (GSTs) (Figure 4A,B). GSTs are a protein superfamily involved in xenobiotic metabolism, frequently acting on compounds modified by cytochrome P450 enzymes [58] (Figure 4C). This protein family catalyzes the conjugation of glutathione to electrophilic compounds, increasing their water solubility for detoxification and excretion [59].

After characterizing the transcriptional changes shared between NRH and NMNH, we next examined transcriptomic changes unique to each precursor (Figure S3A,B). Performing GO term enrichments on the differentially expressed genes revealed a number of precursor‐specific regulations, including increased enrichment for *cellular response to biotic stimulus* for NMNH, while NRH displayed an upregulation of enrichment profiles related to tRNA processes (Figure S3A). Conversely, genes uniquely downregulated by NMNH were enriched for GO terms associated with energy production, such as *oxidative phosphorylation*, whereas NRH enriched GO terms associated with *chromosome segregation* and *mitotic nuclear division*, suggesting a suppression of cell cycle–related pathways (Figure S3B). Together, these data indicate that although reduced NAD^+^ precursors converge on shared detoxification‐related responses, each compound also elicits distinct regulatory signatures, potentially as precursor‐specific metabolic activities or stress‐response profiles.

**FIGURE 5:**
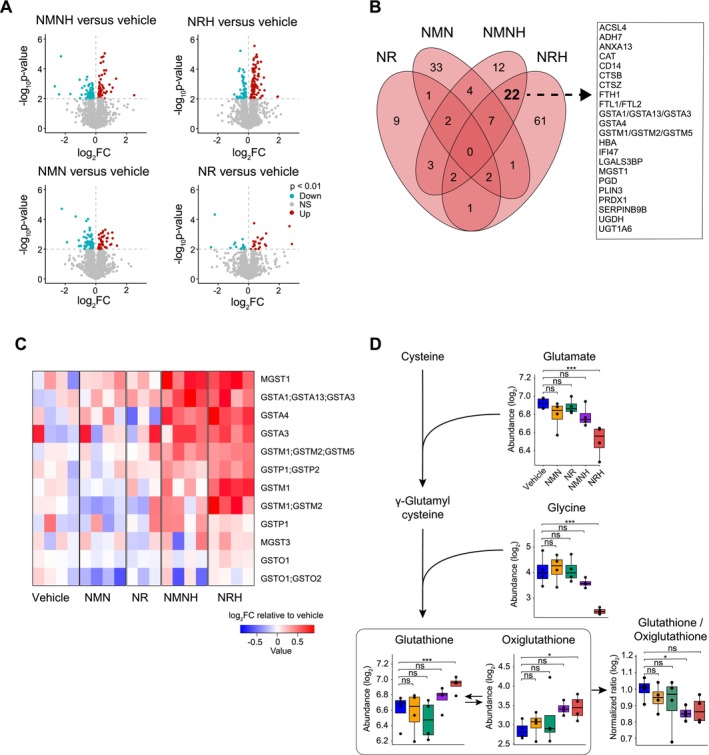
Glutathione‐related proteomic and metabolic changes after NAD^+^ precursor treatment. (A) Volcano plots representing protein abundance changes following NAD^+^ precursor treatment. The *x* axis represents the fold change (log_2_), and the *y* axis represents *p*‐value (−log_10_). Color indicates upregulated (red), downregulated (blue), or not significant (NS) proteins relative to the vehicle group. Dashed lines mark 0 fold change and *p* = 0.01 on the *x* and *y* axes, respectively. (B) Venn diagram of upregulated proteins (*p* < 0.01) following NAD^+^ precursor treatment. Proteins in the intersection between the reduced NAD^+^ precursors, NRH, and NMNH are shown in text box. (C) Heatmap displaying all proteins in the proteomic dataset belonging to the GST family. Color represents the magnitude of upregulation (red) or downregulation (blue), normalized to the vehicle group. (D) Abundance levels of metabolites involved in the biosynthetic pathway of glutathione together with the normalized glutathione/oxiglutathione ratio. Empirical Bayes moderated *t*‐test (two‐sided, unadjusted *p*‐values) was used to determine statistical significance for each metabolite. Normalized redox ratio of log_2_ abundances was calculated relative to the vehicle group and analyzed using one‐way ANOVA. *p < 0.05, **p < 0.01, ***p < 0.001, ns = not significant. Whisker plots display the median, interquartile range (box edges from 25th to 75th percentile), whiskers extending up to 1.5× interquartile range, and individual outliers beyond this range. Source data is available in Table S3.

With the strong effects of reduced NAD^+^ precursors observed at a transcriptomic level, our next step was to explore if similar effects were reflected at a protein level. We performed MS‐based proteomics and were able to annotate 3790 individual proteins or protein groups. In response to the reduced NAD^+^ precursors, we observed a greater number of protein‐level changes compared with the oxidized forms (Figure 5A,B), in line with the metabolomic and transcriptomic results. Importantly, the changes in *Gst* genes observed in the transcriptome data were also reflected in the proteome data, such as shared upregulation of GSTA1/GSTA13/GSTA3, GSTA4, GSTM1/GSTM2/GSTM5, and MGST1 in response to NMNH and NRH (Figure 5B). In fact, 8 out of 12 detected GSTs in the proteome dataset were significantly upregulated (*p* < 0.05) following exposure to either NMNH, NRH, or both (Figure 5C, Figure S4A–L).

Despite increased expression of several GSTs at both transcriptomic and proteomic levels, we did not observe depletion of reduced or oxidized glutathione in most cases (Figure 5D). In fact, NRH supplementation led to a significant increase in both reduced and oxidized glutathione, accompanied by decreased abundance of its precursors, glutamate and glycine. In contrast, the abundance of reduced and oxidized glutathione as well as glutamate and glycine, remained unchanged following exposure to NR, NMN, or NMNH.

Due to the stress‐related response triggered by exposure to reduced NAD^+^ precursors, we next asked whether this reflected impaired mitochondrial integrity. Chronic NAD^+^ deprivation has recently been shown to cause mitochondrial DNA (mtDNA) leakage, suggesting that disturbances in NAD^+^ levels—whether too low or too high—might similarly disrupt mitochondrial function [60]. Using doxorubicin as a positive control, which has previously been shown to induce mtDNA leakage [61], we assessed cytosolic mtDNA levels after NAD^+^ precursor treatment. We did not observe increased mtDNA leakage with either precursor (Figure S5), suggesting that NRH and NMNH do not induce detectable mitochondrial genome instability despite triggering stress‐related signatures.

Overall, these results suggest that reduced NAD^+^ precursors trigger a cellular pseudo‐stress response that occurs in the absence of an actual cellular stressor. As a result, no xenobiotic compound is available for conjugation with glutathione, preventing its depletion.

## 4 Discussion

The reduced forms of NR and NMN, known as NRH and NMNH, have gained considerable attention in recent years as novel NAD^+^ precursors. These compounds exhibit significantly enhanced NAD^+^ boosting capabilities, along with improved circulatory stability compared to the commercially available NAD^+^ precursors NR, and NMN [28, 30]. A number of studies have demonstrated the preclinical therapeutic effects of reduced NAD^+^ precursors, including protection against renal injury [28, 30], aminoglycoside‐induced ototoxicity [62], and metabolic disturbances [63]. In this study, we explored the metabolic (using metabolomics), transcriptomic, and proteomic responses following acute exposure to each precursor in AML12 cells. We confirm that reduced NAD^+^ precursors boost the NAD^+^ metabolome more potently than oxidized precursors. Interestingly, exposure to reduced precursors (NRH or NMNH) also led to a marked increase in the levels of their oxidized counterparts, NR and NMN. This phenomenon has been previously attributed to reverse conversion of NAD^+^ to its precursors when intracellular NAD^+^ levels become excessively high [28, 64]. In addition, a reversible conversion of NAD^+^ to NMN and ATP catalyzed by NMN adenylyltransferase 3 (NMNAT3) has recently been described to occur within mitochondria [65]. Despite the distinct NAD^+^ boosting capacity of NRH and NMNH, the NAD^+^/NADH and NADP^+^/NADPH ratios showed a modest and comparable increase across all precursors, suggesting a tight regulation of the cellular redox state, even under conditions of substantial precursor‐driven NAD^+^ accumulation.

Reduced NAD^+^ precursors imparted far larger transcriptional changes than their oxidized forms. These widespread transcriptional shifts likely mirror the more profound alterations observed at the metabolic level. In particular, NRH treatment led to depletion of glycolytic and TCA cycle intermediates, suggesting a substantial metabolic rewiring that may underlie or contribute to the broad transcriptional response. Metabolic dysfunction, manifested as decreased basal respiration, ATP production, and non‐mitochondrial respirationhas been reported following 24 h of acute NRH exposure [66]. In our study, the indication of energy suppression was unique to NRH, while NMNH treatment in general did not affect energy‐related metabolites. This is in contrast to a study that found decreased abundance of glycolytic and TCA cycle metabolites determined with mass spectrometry following NMNH treatment in HepG2 cells [67].

The response to reduced NAD^+^ precursors inflicted a pseudo‐stress response manifested as an upregulation of the phase II detoxification enzyme family, GSTs, without causing depletion of glutathione or oxiglutathione. This raises the question of whether the cellular effects induced by reduced NAD^+^ precursors will have a superior therapeutic potential than their oxidized counterparts, or if the benefit/risk ratio can tip towards more unfavorable outcomes. For instance, NRH induces a dose‐dependent cytotoxic response in HepG3 cellsat concentrations as low as 100 μM, mediated by increased ROS, mitochondrial superoxide, and oxidized glutathione levels. However, NRH supplementation in HEK293T cells did not lead to such stress responses [66]. Even though we found transcriptional and proteomic indications of redox stress, we did not observe similar changes in glutathione or oxiglutathione levels. In fact, despite the increased expression of GSTs following NMNH and NRH treatment, we found a significant increase in both glutathione and oxiglutathione levels following exposure to NRH. Due to the limitations of metabolomics, which only capture a snapshot of the cellular state, it remains uncertain if glutathione depletion or cellular stress would have been manifested if we had continued the treatment beyond the 24‐h time point. Nonetheless, decreased levels of oxiglutathione have been reported as early as 1 and 4 h in HEK293T and HepG3 cells, respectively [66].

Although reduced NAD^+^ precursors increase NAD^+^ levels more effectively, their stronger metabolic impact may also increase the risk of metabolic stress. The underlying mechanism has not yet been fully elucidated. However, it has been proposed that rapid NAD^+^ accumulation can transiently affect redox balance through concomitant increase of reducing equivalents such as NAD(P)H, ultimately triggering reductive and/or oxidative stress [66]. This increase may also alter the equilibrium of NAD(H)‐ and NADP(H)‐dependent enzymes. At the same time, some of these stress‐related responses may reflect hormesis rather than toxicity, especially since we did not observe indications of mtDNA leakage. Together, this highlights that the superior potency of reduced NAD^+^ precursors may not translate uniformly in vivo, and tissue‐specific metabolic capacity will likely determine whether they confer benefit or harm.

Previous studies have demonstrated that cellular responses to NAD^+^ precursor exposure differ markedly between cell types [28, 30, 66]. As such, a limitation of the current study is the reliance on a single immortalized cell line (AML12) and in vitro conditions, which restricts the generalizability of the findings and may not fully capture the complexity of cell‐ or organism‐level metabolic responses. Another limitation is the use of DMEM as culture media. Although widely used, DMEM may not accurately reflect metabolism compared to more physiologically relevant media, and its non‐physiological nutrient composition could alter baseline and stimulate metabolic activity. Despite these limitations, our findings emphasize the complex picture of boosting NAD^+^ with its precursors.

In summary, this study provides a comprehensive comparative analysis of NAD^+^ precursors in cultured hepatocytes, highlighting their precursor‐dependent transcriptomic, proteomic, and metabolic effects. Our findings suggest that reduced NAD^+^ precursors may have unique therapeutic potentials, influencing cellular metabolism in diverse ways. Conversely, their unique metabolic, transcriptomic, and proteomic effects could also contribute to metabolic stress or toxicity. Future research should explore these differences in vivo and across different cell types to optimize NAD^+^ restoration strategies for metabolic and age‐related diseases.

## Author Contributions

K.T.V., R.Z.‐P., G.E.J., and R.H.H. conceptualized the project and experiments. R.Z.‐P., K.T.V., and S.D. performed cell‐based experiments. K.T.V. and S.D. developed and performed the cytosolic mtDNA determination. R.O. supported with functional experiments. A.J. processed transcriptomic data. K.T.V., B.V.S., M.W., A.J., and G.E.J. developed and performed metabolomic and transcriptomic analyses. B.V.S., M.W., K.T.V., S.R.P., and C.R.J. developed and performed proteomics analyses. K.T.V., R.Z.‐P., G.E.J., and R.H.H. wrote the manuscript with input from all authors.

## Funding

This project has received funding from the European Union's Horizon Europe research and innovation programme under grant agreement No 101073251 (NADIS project). R.Z.P. is supported by a project (PID2023‐147560OA‐I00) from the Ministerio de Ciencia, Innovación y Universidades—Agencia Estatal de Investigación—https://doi.org/10.13039/501100011033 and by the Fondo Europeo de Desarrollo Regional (FEDER, EU).

## Conflicts of Interest

The authors declare no conflicts of interest.

## Supporting information


**Data S1:** fsb271582‐sup‐0001‐Figures.pdf.

**Data S2:** fsb271582‐sup‐0002‐supinfo.csv.

**Data S3:** fsb271582‐sup‐0003‐supinfo.csv.

**Data S4:** fsb271582‐sup‐0004‐supinfo.csv.

## Data Availability

The data that support the findings of this study are available in the Supporting Information of this article.
