# Supplementary material for: Reduced Versus Oxidized NAD + Precursors Drive Distinct Transcriptomic, Proteomic, and Metabolic Profiles in Hepatocytes
Source: FASEB J. 2026 Feb 17;40(4):e71582. doi: 10.1096/fj.202501925R (PMC12911941; doi:10.1096/fj.202501925R)
Supplement: Supplementary file 1 — Data S1: fsb271582‐sup‐0001‐Figures.pdf. [file FSB2-40-e71582-s002.pdf]

Supplementary Figures

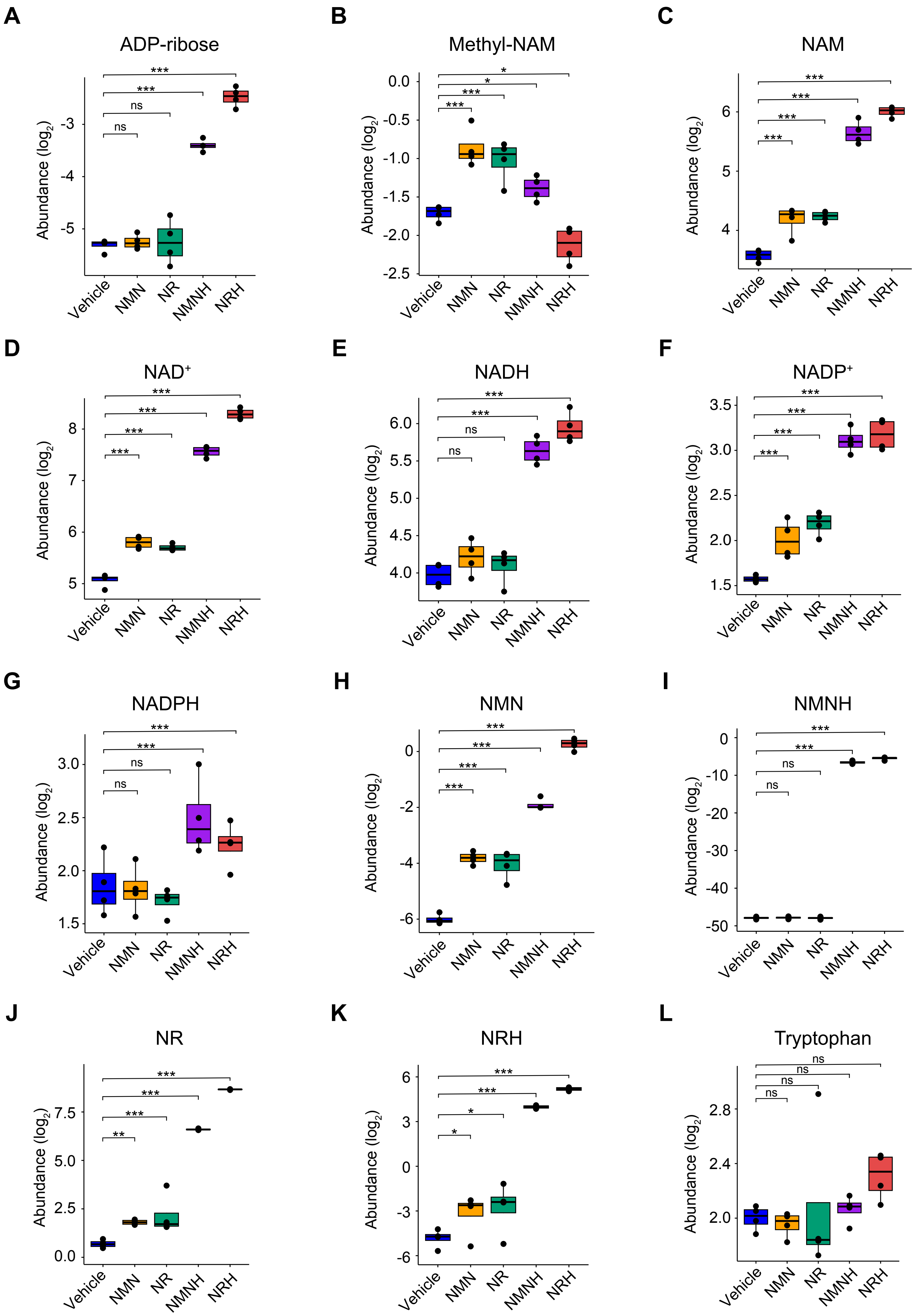

(legend on next page)

Supplementary Figure 1: Abundance levels of NAD^+-related metabolites following NAD^+ precursor treatment. Abundance levels of NAD^+-related metabolites following treatment with NMN, NR, NMNH, NRH (500 μM) or vehicle (PBS) (n = 4) for (**A**) ADP-ribose, (**B**) Methyl-NAM, (**C**) NAM, (**D**) NAD^+, (**E**) NADH, (**F**) $NADP^+$, (**G**) NADPH, (**H**) NMN, (**I**) NMNH, (**J**) NR, (**K**) NRH, (**L**) Tryptophan. Empirical Bayes moderated t-test (two-sided, unadjusted p-values) was used to determine statistical significance, * $p < 0.05$, ** $p < 0.01$, *** $p < 0.001$, ns = not significant. Whiskers plots display the median, interquartile range (box edges from 25th to 75th percentile), whiskers extending up to 1.5 × interquartile range, and individual outliers beyond this range. Source data is available in supplementary table 1.

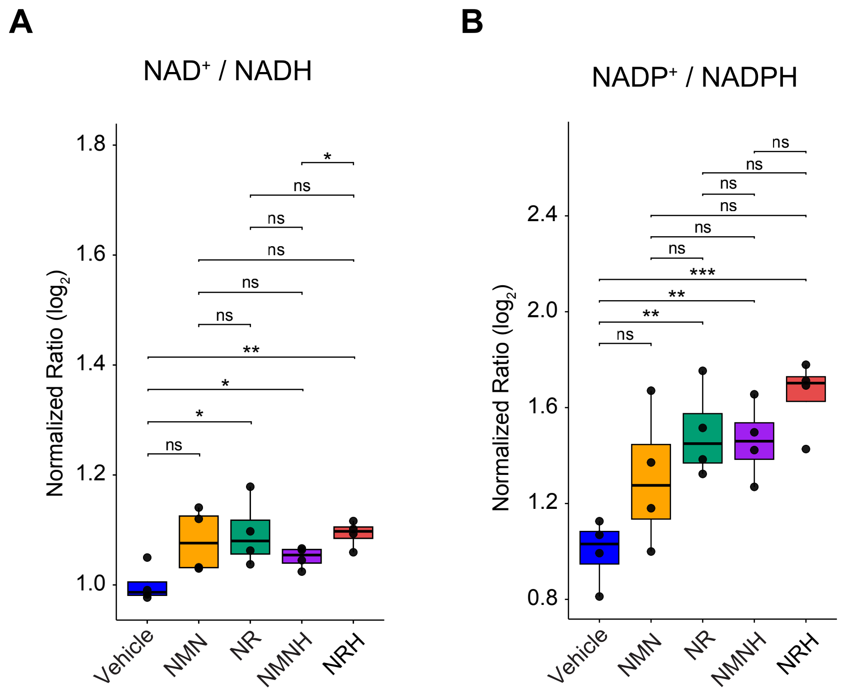

Supplementary Figure 2: Normalized redox ratios following NAD$^+$ precursor treatment. Normalized redox ratios were calculated relative to the vehicle group of redox pair: **(A)** NAD$^+$ / NADH and **(B)** NADP$^+$ / NADPH (n = 4). One-way analysis of variance (ANOVA) was used to determine statistical significance, * $p < 0.05$, ** $p < 0.01$, *** $p < 0.001$, ns = not significant. Whiskers plots display the median, interquartile range (box edges from 25th to 75th percentile), whiskers extending up to 1.5 × interquartile range, and individual outliers beyond this range. Source data is available in supplementary table 1.

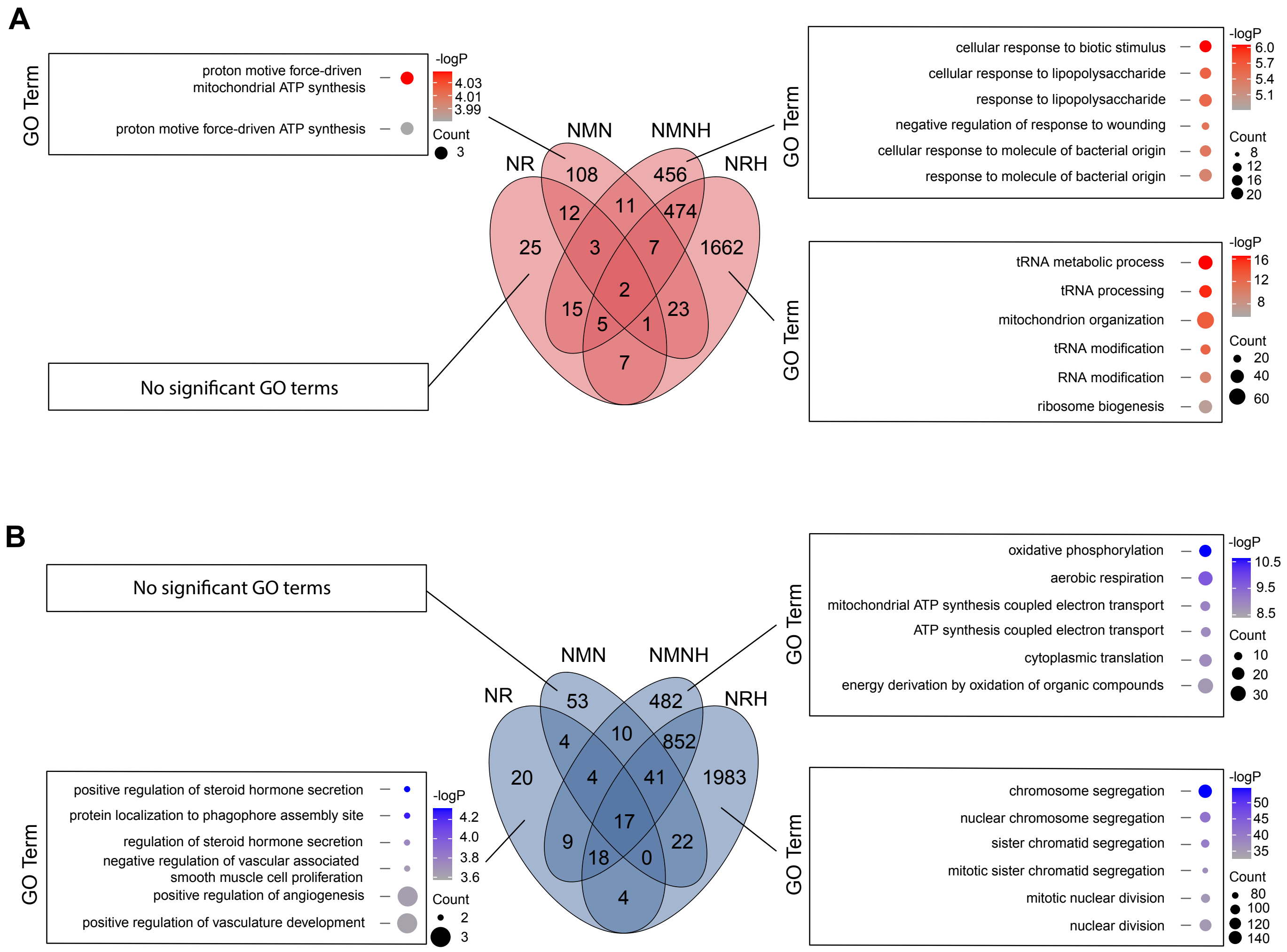

Supplementary Figure 3: NAD$^+$ precursor-specific enriched GO terms. Venn diagrams of **(A)** upregulated (red) and **(B)** downregulated (blue) genes following NAD$^+$ precursor treatment ($p < 0.01$). Gene ontology (GO) term analysis was applied to each of the unique sections of each NAD$^+$ precursor using a p-value cut-off of 0.05.

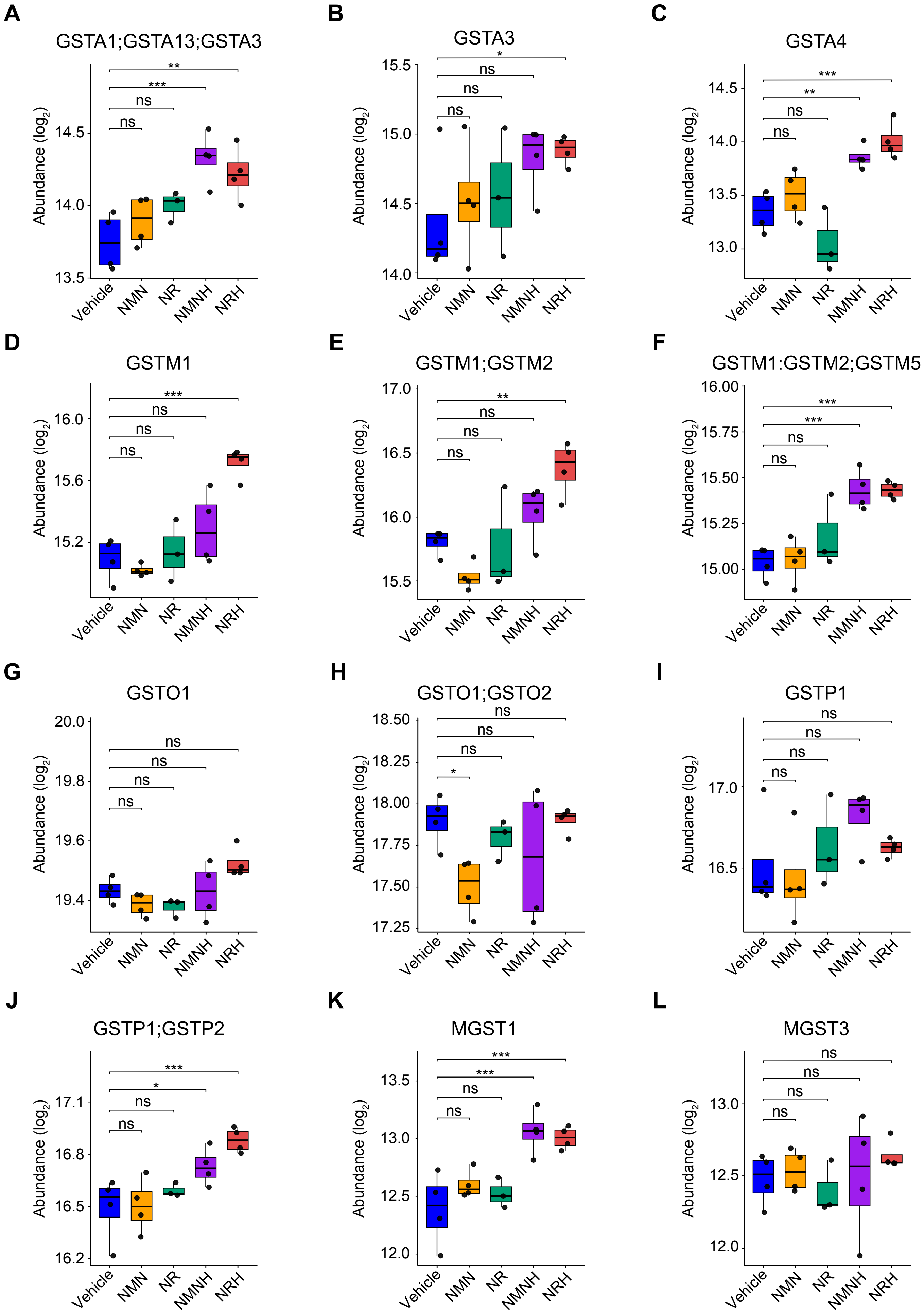

(legend on next page)

Supplementary Figure 4: Abundance levels of proteins belonging to the GST family following NAD^+ precursor treatment. Abundance levels of GSTs following treatment with NMN, NR, NMNH, NRH (500 μM) or vehicle (PBS) (n = 3-4) for (**A**) GSTA1;GSTA13;GSTA3, (**B**) GSTA3, (**C**) GSTA4, (**D**) GSTM1, (**E**) GSTM1;GSTM2, (**F**) GSTM1:GSTM2;GSTM5, (**G**) GSTO1, (**H**) GSTO1;GSTO2, (**I**) GSTP1, (**J**) GSTP1;GSTP2, (**K**) MGST1, (**L**) MGST3. Empirical Bayes moderated t-test (two-sided, unadjusted p-values) was used to determine statistical significance, * $p < 0.05$, ** $p < 0.01$, *** $p < 0.001$, ns = not significant. Whiskers plots display the median, interquartile range (box edges from 25th to 75th percentile), whiskers extending up to 1.5 × interquartile range, and individual outliers beyond this range. Source data is available in supplementary table 3.

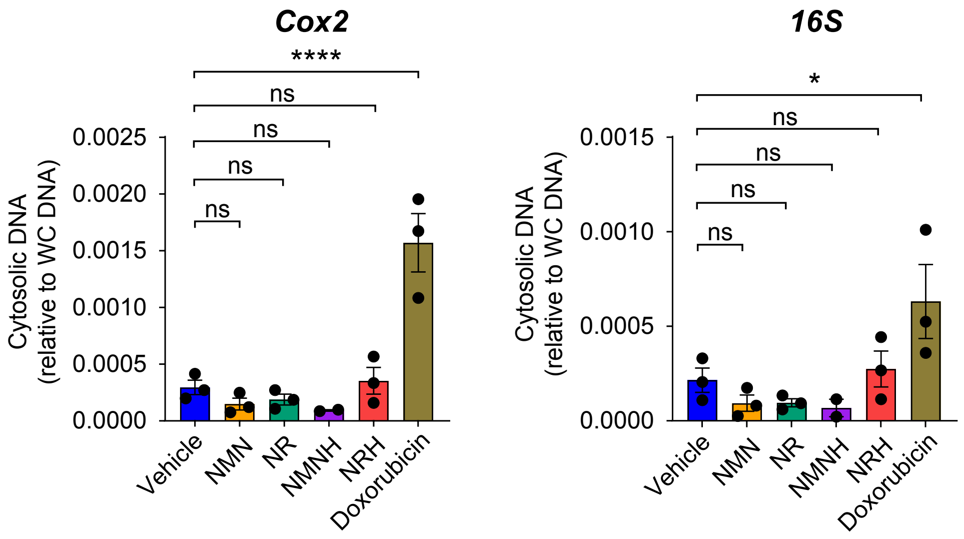

Supplementary Figure 5: Leakage of mitochondrial DNA into the cytosol following NAD$^+$ precursor treatment. Levels of cytosolic mitochondrial DNA in AML12 cells following treatment with NMN, NR, NMNH, NRH (500 μM), doxorubicin (1 μM) or vehicle (PBS) (n = 2-3) using qPCR. The panels indicate cytosolic mitochondrial DNA normalized to individual whole cell (WC) DNA. Data are presented as mean ± SEM, with n representing the number of biological replicates. p-values are calculated using one-way analysis of variance (ANOVA). *$p < 0.05$, ****$p < 0.0001$.
